# Comparative linkage analysis and visualization of high-density oligonucleotide SNP array data

**DOI:** 10.1186/1471-2156-6-7

**Published:** 2005-02-15

**Authors:** Igor Leykin, Ke Hao, Junsheng Cheng, Nicole Meyer, Martin R Pollak, Richard JH Smith, Wing Hung Wong, Carsten Rosenow, Cheng Li

**Affiliations:** 1 Department of Biostatistics, Harvard School of Public Health, Boston, MA 02115, USA; 2 Department of Biostatistical Science, Dana-Farber Cancer Institute, 44 Binney Street, Boston, MA 02115, USA; 3 Department of Computer Science, University of Illinois at Chicago, Chicago, IL 60607, USA; 4 Molecular Otolaryngology Research Labs, University of Iowa, Iowa City, IA 52242, USA; 5 Department of Medicine, Brigham and Women's Hospital and Harvard Medical School, Boston, MA 02115, USA; 6 Department of Statistics, Stanford University, Stanford, CA, 94305, USA; 7 Affymetrix Inc., 3380 Central Expressway, Santa Clara, CA 95051, USA

## Abstract

**Background:**

The identification of disease-associated genes using single nucleotide polymorphisms (SNPs) has been increasingly reported. In particular, the Affymetrix Mapping 10 K SNP microarray platform uses one PCR primer to amplify the DNA samples and determine the genotype of more than 10,000 SNPs in the human genome. This provides the opportunity for large scale, rapid and cost-effective genotyping assays for linkage analysis. However, the analysis of such datasets is nontrivial because of the large number of markers, and visualizing the linkage scores in the context of genome maps remains less automated using the current linkage analysis software packages. For example, the haplotyping results are commonly represented in the text format.

**Results:**

Here we report the development of a novel software tool called CompareLinkage for automated formatting of the Affymetrix Mapping 10 K genotype data into the "Linkage" format and the subsequent analysis with multi-point linkage software programs such as Merlin and Allegro. The new software has the ability to visualize the results for all these programs in dChip in the context of genome annotations and cytoband information. In addition we implemented a variant of the Lander-Green algorithm in the dChipLinkage module of dChip software (V1.3) to perform parametric linkage analysis and haplotyping of SNP array data. These functions are integrated with the existing modules of dChip to visualize SNP genotype data together with LOD score curves. We have analyzed three families with recessive and dominant diseases using the new software programs and the comparison results are presented and discussed.

**Conclusions:**

The CompareLinkage and dChipLinkage software packages are freely available. They provide the visualization tools for high-density oligonucleotide SNP array data, as well as the automated functions for formatting SNP array data for the linkage analysis programs Merlin and Allegro and calling these programs for linkage analysis. The results can be visualized in dChip in the context of genes and cytobands. In addition, a variant of the Lander-Green algorithm is provided that allows parametric linkage analysis and haplotyping.

## Background

The oligonucleotide Mapping 10 K arrays [1] have been used for linkage analysis [2-4] and their advantages in genome coverage and information content compared to microsatellite-based assays has been demonstrated. The array contains 11,550 SNPs with an average heterozygosity rate of 0.32 and an average marker distance of 0.31 cM. However, the commonly used multi-point linkage analysis software packages such as GeneHunter [5,6] and Merlin [7] are command-line programs and it is not straightforward to find genes in the regions of high linkage scores. In addition, the haplotyping results are represented commonly in a text format without any gene context.

Here we report the development of a new software tool called CompareLinkage that can be used for automated conversion of Mapping 10 K genotype data into the "Linkage" format for linkage analysis in Merlin, GeneHunter and Allegro [8]. In addition the program can convert the pedigree information and SNP marker information into the "Linkage" format. After performing the linkage analysis using one or more of these programs, the CompareLinkage software can export the linkage score information into the dChip software [9-11] to visualize the results within a chromosome window. In addition, we implemented a variant of the Lander-Green [5,12] algorithm into the dChipLinkage module to analyze pedigrees with up to 18 bits (bits = 2n-f ; with n = number of non-founders and f = number of founders) using the parametric linkage analysis method. We are currently testing and validating the implementation of the algorithm which will be described in detail elsewhere. The linkage score curves, genotypes and haplotypes are graphically displayed in a dChip chromosome window which has the genes, cytoband and SNP marker information included. Together the CompareLinkage and dChip software programs provide for the first time a graphical user interface (GUI) and an automated procedure for comparative linkage analysis utilizing three commonly used linkage software programs.

## Implementation

### The CompareLinkage software for comparative linkage analysis using Merlin and Allegro

To analyze large pedigrees rapidly and to compare the linkage analysis results of different software packages, we developed a software tool called CompareLinkage to automate the following processes: (1) Converting of Affymetrix Mapping 10 K genotype data, pedigree files and marker information into the "Linkage" format [13], and detecting and fixing incompatibilities in pedigree genotypes. The input genotype text file for CompareLinkage can be a single text file containing genotypes for each sample or a combined text file as exported by the Affymetrix GDAS 3.0 software. (2) Automatically calling the software packages Merlin and Allegro for linkage analysis and converting the analysis results (LOD or non-parametric linkage (NPL) scores) into the input files for dChip to visualize the results in the context of genes and cytobands. (3) The SNP genotype data in the "Linkage" format can be converted into the dChip input files (genotype, pedigree and marker information files) to perform parametric linkage analysis by dChipLinkage. All steps are discussed in detail at the CompareLinkage software manual provided on the software website. All these functionalities are useful for cross-validation of linkage results and to identify concordance and discordances between different linkage analysis programs as well as between parametric and non-parametric linkage results.

**Figure 1:**
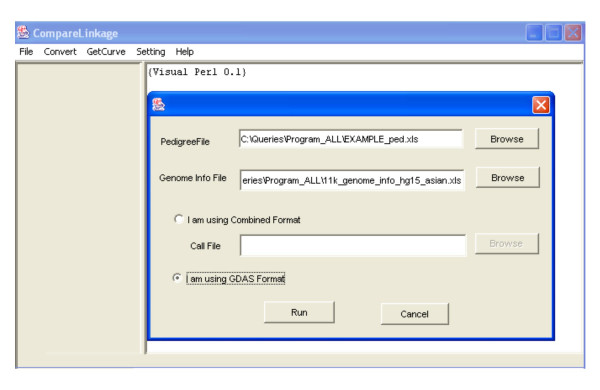
The CompareLinkage GUI dialog for choosing pedigree, genome information and genotype call files.

**Figure 2:**
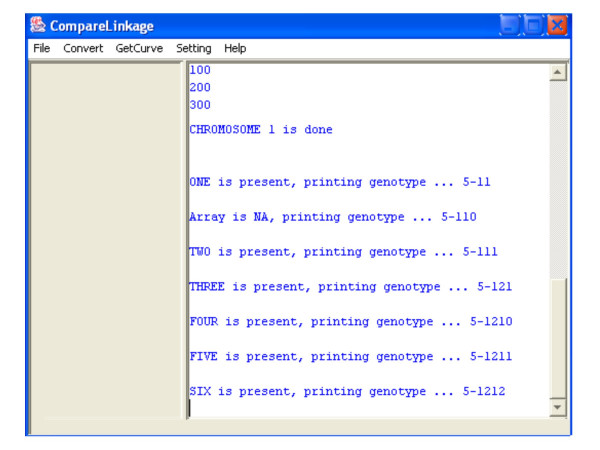
The intermediate output of the CompareLinkage GUI.

A graphical user interface (GUI) for Windows was also implemented in Java. In this GUI users are allowed to set their own working directory and the location of the Perl interpreter through the "Setting" menu. CompareLinkage's functions of converting file formats and getting dChip input files are incorporated through the "Convert" and "GetCurve" menu (Figure 1). Since computing is usually time-consuming, the code of calling the Perl program is executed in separate thread to provide better interaction. The output of the Perl program can be viewed in the message window (Figure 2).

### The dChipLinkage software module

The Affymetrix Mapping 10 K array CEL files and genotype TXT files can be imported into dChip and visualized along cytobands and genes as previously reported [9,11]. The information of the SNPs such as their genetic and physical distance and allele frequencies from three ethnic groups (Asian, African American and Caucasian) is obtained from the Affymetrix website [14] and converted into the genome information files for dChip. The information of the reference genes and cytobands is obtained from the UCSC genome bioinformatics database [15] for the matching human genome assembly (hg12 or hg15) of the SNP information, and is organized into the refGene and cytoband file provided with dChip.

**Figure 3:**
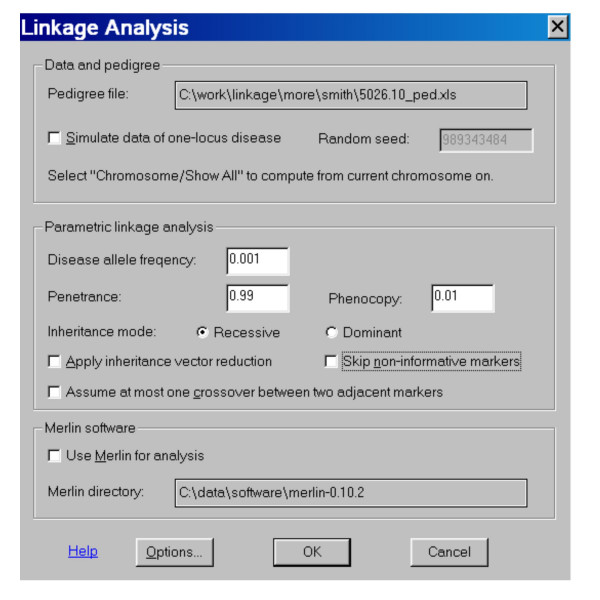
The dChipLinkage dialog for specifying linkage analysis parameters.



We implemented a variant of the Lander-Green [5,6,16] algorithm in the dChipLinkage module of dChip to perform multipoint parametric linkage analysis and compute a LOD score at each SNP position. Disease allele frequencies, penetrance information and phenocopy information for dominant and recessive disease models can be selected by the user through a dialog (Figure 3). The Mendelian genotype errors inconsistent with parental genotypes are detected and set to missing genotypes. To handle other genotyping errors or wrongly mapped SNP markers, we assume a conservative genotyping error rate of 0.01 [1] (user adjustable) and regard observed genotypes as phenotypes in the likelihood computation [17]. As a result, the computation of the probability of the observed genotype data at one marker given an inheritance vector *v *involves the summation over all the possible real genotypes (or equivalently the founder allele configurations):

where F_i _represents the *i*th of all the possible founder allele configurations and is independent of *v*. *P*(real genotypes *i *| *v*, F_i_) is 1 since an inheritance vector and founder allele configuration uniquely determines the real genotypes, and *P*(observed genotypes | real genotypes *i*) involves comparing the real genotype and observed genotype for all the individuals and multiplying the probability by the error rate of 0.01 (default value) for each disagreement and 0.99 for each agreement. We also use the matrix-vector multiplication algorithm and bit reduction due to founder phase symmetry described in [16], and the founder allele factoring technique reported in [6,17] to speed up the computation of single-locus and accumulative likelihood vectors as well as the likelihood vector of disease phenotypes.

We use the forward-backward computation in the Lander-Green algorithm to obtain the marginal probability distribution of inheritance vector at each SNP marker position given the data of all the markers on a chromosome. In addition the most likely inheritance vector at each marker given the genotype data of all the markers on this chromosome is calculated [6]. Conditioned on the most likely inheritance vector at a marker and the observed genotype data, we can find the most likely founder allele configurations. When there are competing inheritance vectors with the same largest marginal probabilities at a marker, we select the one with fewer crossover events from the last marker since the distance between adjacent markers are small (average 300 kb) and it is therefore less likely to have multiple crossover events between two markers in a pedigree [7]. Together these procedures give the haplotyping results of the pedigree data. dChipLinkage visualizes the haplotyping result in either the haplotype view or the ordered genotype view.

## Results

### The comparative linkage analysis using Merlin, GeneHunter, Allegro and dChipLinkage

**Figure 4:**
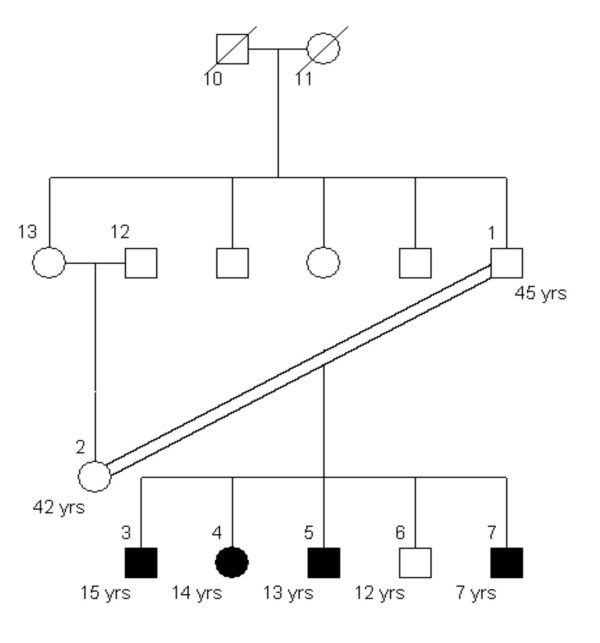
The pedigree structure of family 5026.10. The PED 4.2 software is used to draw the pedigrees.

**Figure 5:**
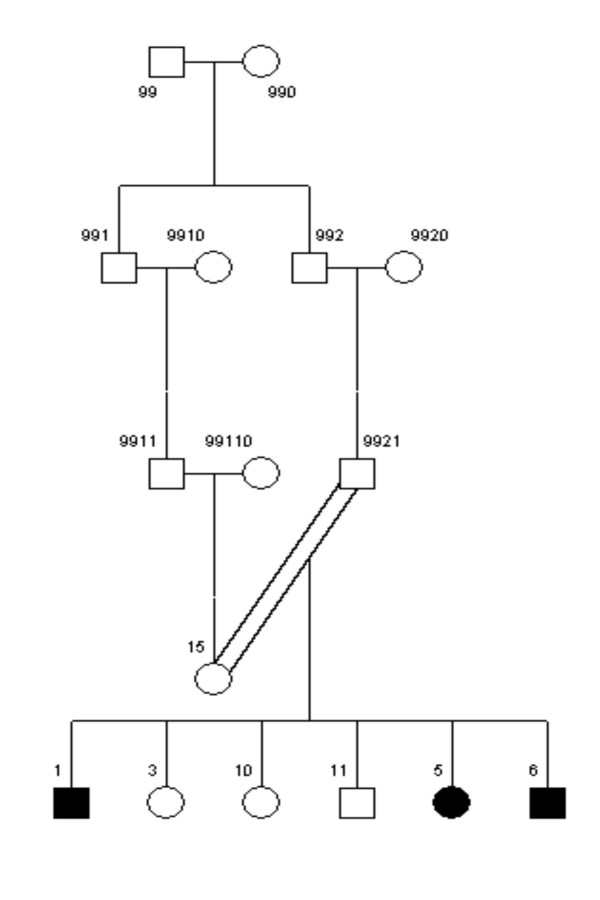
The pedigree structure of family CR

**Figure 6:**
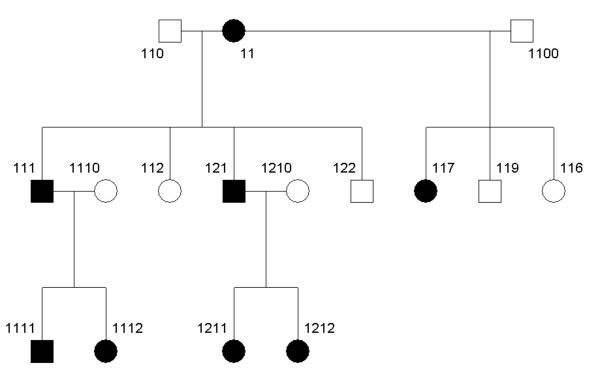
The pedigree structure of family ER.

CompareLinkage can format Affymetrix Mapping 10 K SNP genotype output files and genotype files into the "Linkage" format and convert genome information and pedigree files into the formats suitable for Merlin (Version 0.10.2), GeneHunter (Version 2.1) and Allegro (Version 1.2). CompareLinkage removes all non-informative markers and calls the PedCheck software [18] to detect genotype incompatibilities using the pedigree information. A Mendelian genotype inconsistency at a SNP is handled by setting the genotype of this SNP in all the individuals to missing. For the analysis in GeneHunter, overlapping segments of large chromosomes are prepared, with each segment containing 150 or fewer markers with 75 markers in common between adjacent segments. Linkage scores are computed as the mean of two scores for the same marker from the two overlapping fragments. We ran genome-wide linkage analysis using all the three software packages and dChipLinkage for the 10 K SNP genotype data of three families: 5026.10 (Figure 4; autosomal recessive non-syndromic deafness disease, 13 bits, Asian), CR (Figure 5; recessive, 17 bits, Asian) and ER (Figure 6; dominant, 17 bits, Caucasian). For the parametric analysis, we use a disease frequency of 0.001, a penetrance value of 0.99 and a phenocopy of 0.01 for all the families and all the software packages. For GeneHunter and Allegro we ran both nonparametric and parametric analysis. For Merlin, the NPL_all statistic is computed. The allele frequencies are calculated based on the actual genotype data in each family. The LOD score or NPL score are computed at the position of the SNP makers. After running the analysis for all chromosomes, the two chromosomes with the largest LOD scores were selected from each pedigree and compared below.

**Figure 7:**
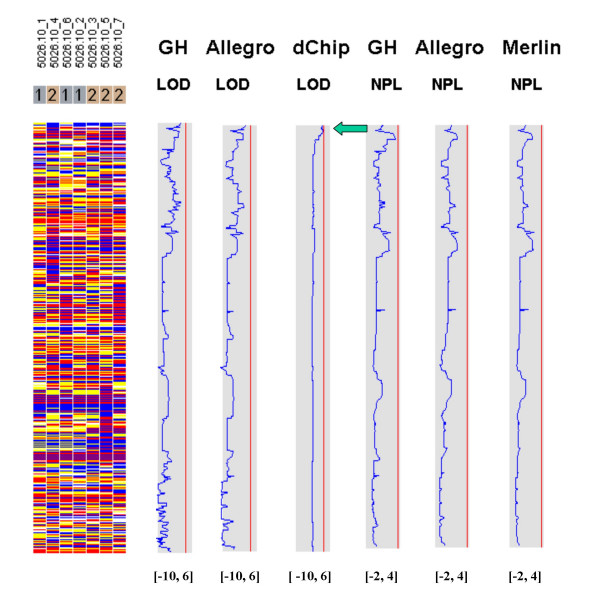
The comparative linkage results of the chromosome 1 of the family 5026.10 using CompareLinkage and dChipLinkage. The genotype calls are displayed on the left in yellow (AB), red (AA) and blue (BB), with SNPs on rows and samples on columns. The sample names and the disease status (1 = Unaffected and 2 = Affected) are displayed on the top. The linkage scores of different software are displayed on the right in the shaded box. The lower and upper limits of the shaded box (such as [-10, 6]) are in the brackets on the bottom of the curve. The red vertical line indicates the threshold of 3.0 for LOD scores and 3.7 for NPL scores. This line is user adjustable.

**Figure 8:**
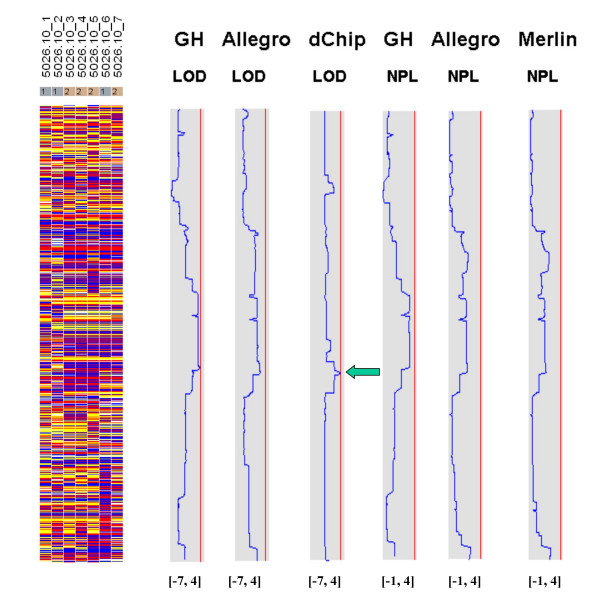
The comparative linkage results of the chromosome 3 from the family 5026.10. The figure format is the same as Figure 7.

**Figure 9:**
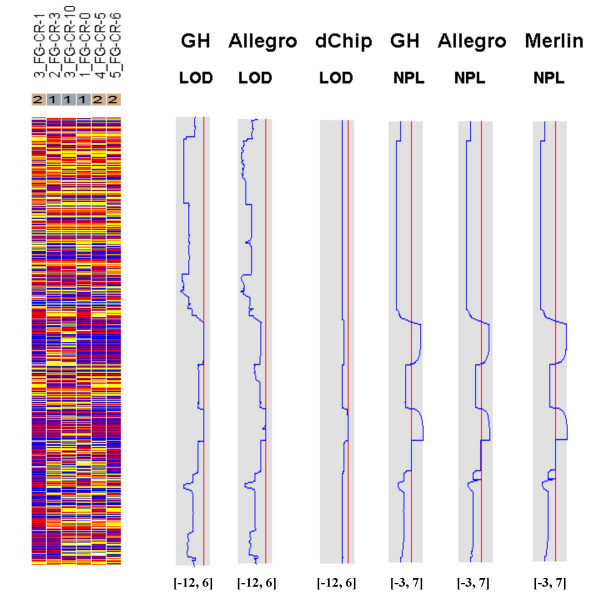
The comparative linkage results of the chromosome A of the family CR.

**Figure 10:**
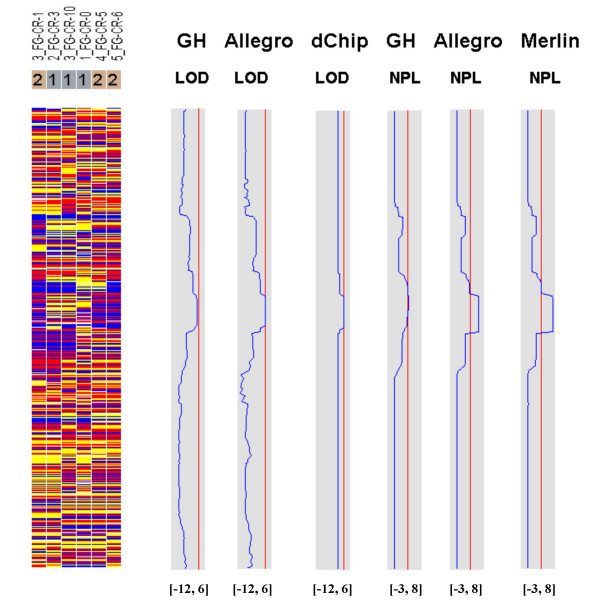
The comparative linkage results of the chromosome B of the family CR.

**Figure 11:**
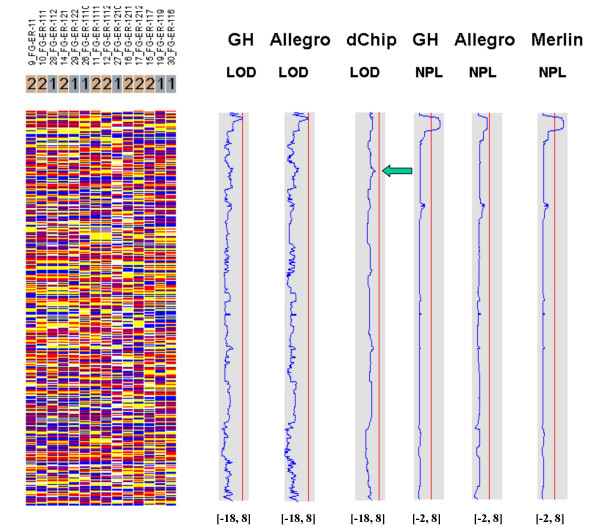
The comparative linkage results of the chromosome A of the family ER.

**Figure 12:**
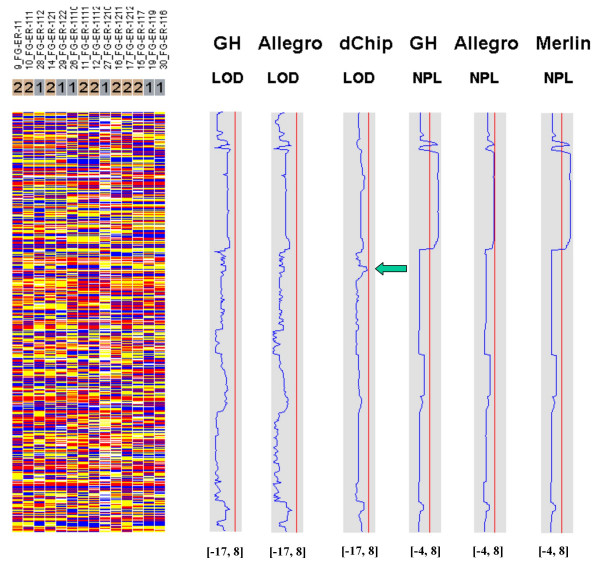
The comparative linkage results of the chromosome B of the family ER.

Figures 7, 8, 9, 10, 11, 12 show the comparative LOD score and nonparametric score plots in dChip for these chromosomes analyzed with GeneHunter, Merlin, Allegro and dChipLinkage. The vertical red line in the figures indicates the significance threshold and is set to 3 for parametric analysis (LOD scores) and to 3.7 for non-parametric analysis (NPL score) based on statistical significance recommended by Lander and Kruglyak [19]. The linkage scores largely agree with each other in the regions with significant LOD/NPL scores. GeneHunter, Merlin and Allegro detect the peaks in the chromosome 1 and 3 of the consanguineous family 5026.10 but compute lower LOD scores than dChipLinkage (indicated by arrows in Figure 7 and 8). For another consanguineous family CR with a recessive disease, all software packages detect similar peak regions in the two chromosomes denoted as A and B (Figure 9 and 10). For the family ER with a dominant disease, dChipLinkage computes similar overall patterns but reports a possible sporadic and non-significant peak (LOD < 1.6) in each chromosome (indicated by arrows in Figure 11 and 12).

### Linkage analysis and visualization using dChipLinkage

**Figure 13:**
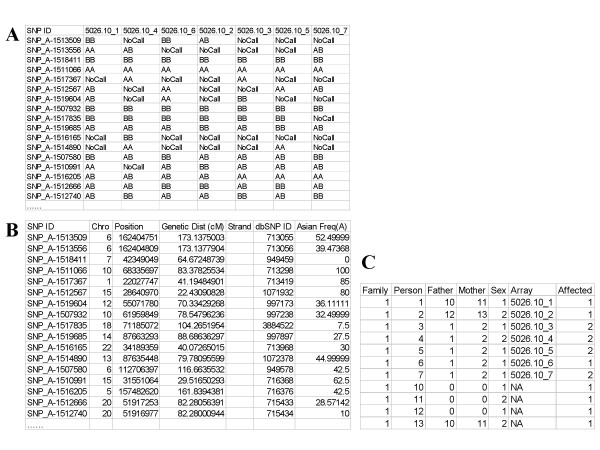
The genotype file (A), genome information file (B) and pedigree file (C) used by dChipLinkage for analysis.

To do parametric linkage analysis in dChipLinkage, a pedigree file is needed (Figure 13C). The file is similar to the standard pedigree file format but has an additional "Array" column matching each individual in the pedigree file to the corresponding genotype information in the genotype file through array names (the header line in Figure 13A). The data importing and analysis steps are:

1. Open dChip.

2. Select the ***Analysis ***menu and the ***Get External Data ***function to read in the genotype file in the text format (Figure 13A).

3. Select the genome information file downloaded from the dChip website (Figure 13B). This file is provided in three versions, each containing the SNP information like TSC SNP ID and genetic map locations but having different allele frequencies for each of the three ethnic groups (Asian, Caucasian and African Americans).

4. Select the ***Analysis ***menu and the ***Chromosome ***function to display the genotype calls, genes and cytobands along the chromosome

5. After the program has displayed the genotype data, select the ***Chromosome ***menu and the ***Linkage ***function to start the dChipLinkage module (Figure 3). Specify the pedigree file (Figure 13C) and other linkage parameters. Depending on whether the dChip "***Chromosome View***" displays one or all chromosomes, the linkage analysis will be performed for one or all chromosomes accordingly. For the analysis of the 5026.10 family, the recessive disease model is assumed, and a penetrance of 0.99, phenocopy of 0.01, disease allele frequency of 0.001 and a SNP marker error rate of 0.01 are used. The SNP allele frequencies in the genome information file are used and truncated to values between 0.001 and 0.999. This family has 13 bits and it takes about 20 minutes for the whole genome linkage analysis.

**Figure 14:**
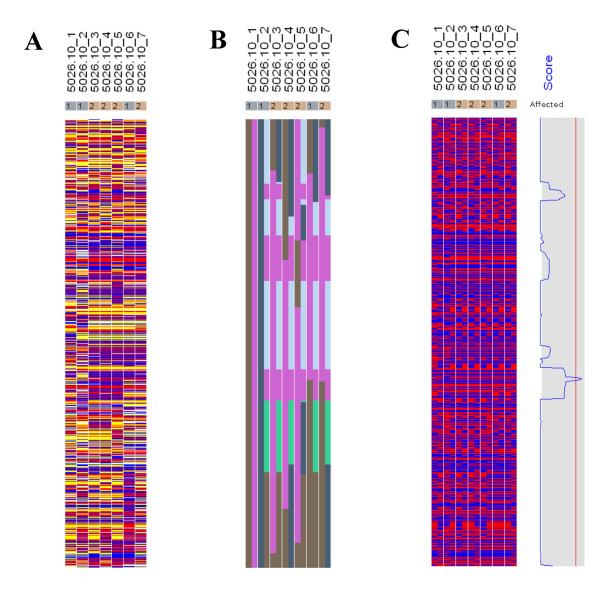
(A) In the genotype view, the red, blue, yellow and white colors represent genotype call AA, BB, AB and No Call. (B) The inferred haplotypes indicating ancestor origins are displayed in correspondence to the genotype view. The different colors represent distinct founder chromosomes. For each individual (column), the father allele haplotype is displayed on the left and mother allele haplotype on the right. (C) In the ordered genotype view, the red and blue colors represent the A and B genotype of father allele (left) and mother allele (right) in each individual (column). The LOD score curve is displayed in the shaded box on the right. The left boundary and right boundary of the box represent value of -2 and 3, and the red vertical line represents 2.

**Figure 15:**
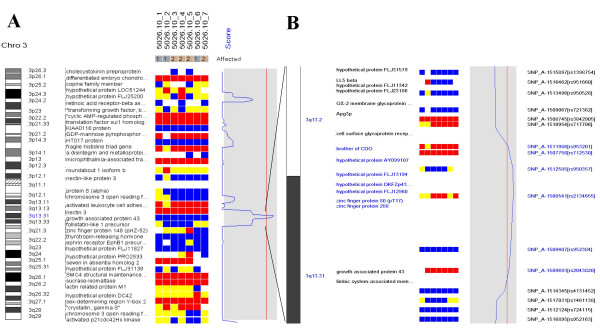
(A) The peak LOD score region is enlarged and displayed proportionally to real chromosomal distance in the context of genes and cytobands. LOD score peaks are shown at the q-arm of chromosome 3 (114.18 -117.00 Mb, maximal LOD = 2.77). The shaded curve region has the same range as Figure 14. (B) A enlarged view of the peak region with more details of the individual SNPs and genes. The transcription starting site of the genes are used to display their positions.

Using dChipLinkage to analyze the 5026.10 family, we were able to identify a region on the chromosome 1 (Cytogenetic region: 1p36.32 – 1p36.22) with LOD scores of greater than 2.3 (Figure 7, indicated by arrow). The most interesting gene in this region is ESPIN, which has previously been shown to be involved in deafness in mice [20] and two frameshift mutations in the gene have just recently been associated with deafness in two consanguineous families [21]. Sequence analysis of the locus revealed that the parents (the individual 1 and 2 in Figure 4) and the unaffected child (the individual 6) are heterozygous for the insertion mutation and the affected children are homozygous (data not shown). In addition a novel locus with a maximum LOD score of 2.77 was identified on the chromosome 3 (Figure 8, indicated by arrow). The peak region on the chromosome 3 is about 2 Mb wide (Figure 14C). Using the GeneHunter software, we compute a maximum expected LOD score of 2.78 for this family under the specified parameters. Therefore we extract the most linkage information based on the dense SNP markers in this region. Figure 14 shows the LOD score curve together with genotype calls, inferred haplotypes and ordered genotypes based on haplotyping. In Figure 15 the results are presented in the context of cytobands and genes. The ***Chromosome/Export SNP data ***function can also export the text information of the SNPs, genes and cytobands in the region with linkage scores exceeding the threshold.

**Figure 16:**
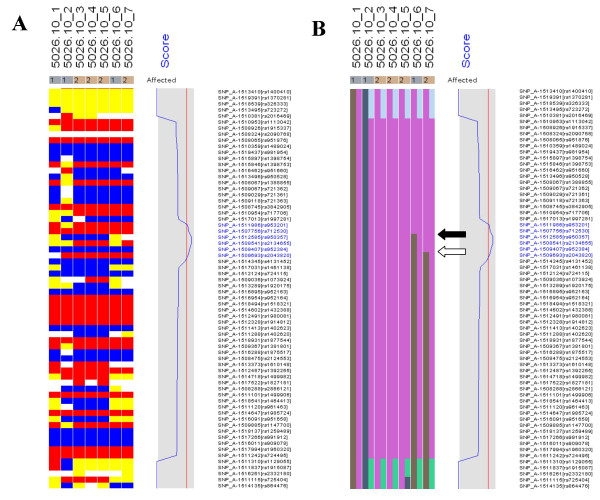
The genotypes (A) and inferred haplotypes (B) from family 5026.10 on the peak score region of chromosome 3 are shown (for more details see the legend in Figure 14). In the peak LOD score region all the affected children (3, 4, 5 and 7) inherited the same ancestral allele in the consanguineous family and the unaffected child (6) inherited two different ancestral alleles.

After the linkage computation is finished, the inferred haplotype information can be visualized. In the haplotype view (Figure 14 and 16), one can view the inference on how the founder chromosomes are crossed over and inherited by the descendants. The different colors represent distinct founder chromosomes, and for each individual, the father allele haplotype is displayed on the left and mother on the right. Since a pedigree contains no phase information of the founders [6], in the linkage computation we can assume that one child of each founder always inherits the whole grandfather-descent chromosome. This assumption does not affect the LOD score computation but reduces the number of bits in the Lander-Green algorithm by the number of founders and consequently reduces the analysis time. This is the reason that in Figure 14B the individual 1 has both father and mother haplotypes in pure color and individual 2 has only the father haplotype in pure color. By inspection of the observed genotype and the inferred haplotypes (Figure 16), one can see that only in the peak LOD score region all the affected children (individual 3, 4, 5 and 7) are homozygous and that the unaffected child (individual 6) is heterozygous. All the affected individuals share two copies of the identical chromosome segment (the pink color between the two arrows) presumably containing the disease locus. By two very close crossover events respectively in individual 6 (indicated by the black arrow) and individual 7 (indicated by the white arrow), the LOD score implicates the possible disease gene in a 2 Mb region and one can easily search the physical map for candidate disease genes in this region in the dChip chromosome view (Figure 15).

## Discussion and conclusions

We have developed the CompareLinkage software for easy comparison and analysis of genotype datasets with common multi-point linkage analysis software programs. It provides functions such as automated data formatting and the calling of linkage analysis software programs to facilitate comparative linkage analysis. The results can be visualized in a chromosome window in the context of genes, cytobands and SNPs in dChip's user friendly graphical interface. The linkage scores of other linkage software packages can be saved into the dChip score file format through CompareLinkage and viewed in the dChip chromosome viewer. This provides the interface to view other computed statistics such as linkage disequilibrium scores along the chromosomes. We have also implemented a variant of the Lander-Green algorithm as the dChipLinkage module for parametric linkage analysis of small pedigrees. It can analyze all chromosomes for families with up to 18 bits within one hour on a PC with one gigabyte memory. This is useful for recessive and consanguineous families whose bits are often small.

The comparison analysis of three Mapping 10 K array data sets show similar results in regions with significant LOD scores across all the four software packages. The regions with concordant LOD/NPL scores should provide more confidence in the candidate disease loci. However, there are clear differences in isolated regions. This emphasizes the challenge of a comparative analysis using different linkage algorithm implementations. We hypothesize that the differences between the software programs in peak locations are attributable to:

1. The specific algorithm implementation in each program.

2. The difference between parametric – and non-parametric analysis.

3. The existence of undetected genotype errors in the data sets which could falsely deflate LOD scores [17,22]. dChipLinkage uses an error model to automatically handle genotype errors and avoid sporadic LOD score peaks due to undetected non-Mendelian errors, and results in a smoother LOD curve as seen in Figure 7, 8, 9, 10, 11, 12. However, this error handling algorithm involves more iterations and increases the computation time. There are further techniques to reduce the memory and time requirement of the Lander-Green algorithm [7,8,23,24]

In light of the discordance between the results from common linkage software packages and from dChipLinkage, we will validate dChipLinkage implementation using additional datasets and the CompareLinkage software.

In summary, the CompareLinkage and dChipLinkage software automate the comparative linkage analysis and visualization using multiple software packages. With these tools users will be able to increase their confidence in candidate regions and can use the visualization tools to explore the disease associated genome regions.

## Availability and requirements

Project name: The CompareLinkage software and the dChipLinkage software module

Project home page: 

Operating system(s): Windows (dChipLinkge); Windows (CompareLinkage and its graphical interface), Unix (CompareLinkage command line version)

Programming language: Visual C++ 6.0 (dChipLinkge); Perl and Java (CompareLinkage software)

Other requirements: None

License: None.

Any restrictions to use by non-academics: No restrictions

## Authors' contributions

CR, CL and WHW conceived of the study, and participated in its design and coordination. NM and RJHS generated the 5026.10 family data, and MP generated the CR and ER family data. IL implemented the CompareLinkage software and performed the comparative analysis using multiple linkage analysis software packages. JC implemented its graphical user interface (GUI). CL implemented the dChipLinkage module. KH participated in the design and analysis of the study. IL, CR and CL drafted the manuscript. All authors read and approved the final manuscript.
